# Advanced intraoperative MRI in pediatric brain tumor surgery

**DOI:** 10.3389/fphys.2023.1098959

**Published:** 2023-04-13

**Authors:** Pien E. J. Jellema, Jannie P. Wijnen, Alberto De Luca, Henk J. M. M. Mutsaerts, Iris V. Obdeijn, Kirsten M. van Baarsen, Maarten H. Lequin, Eelco W. Hoving

**Affiliations:** ^1^ Department of Pediatric Neuro-Oncology, Princess Máxima Centre for Pediatric Oncology, Utrecht, Netherlands; ^2^ Centre for Image Sciences, University Medical Centre Utrecht, Utrecht, Netherlands; ^3^ Department of Neurology, University Medical Center Utrecht, Utrecht, Netherlands; ^4^ Department of Radiology and Nuclear Medicine, Amsterdam UMC Location Vrije Universiteit Amsterdam, Amsterdam, Netherlands; ^5^ Amsterdam Neuroscience, Brain Imaging, Amsterdam, Netherlands; ^6^ Department of Neurosurgery, University Medical Centre Utrecht, Utrecht, Netherlands; ^7^ Department of Radiology, University Medical Centre Utrecht, Utrecht, Netherlands

**Keywords:** intraoperative MRI, advanced MRI, surgical anatomy, postoperative changes, pediatric brain tumor patients

## Abstract

**Introduction:** In the pediatric brain tumor surgery setting, intraoperative MRI (ioMRI) provides “real-time” imaging, allowing for evaluation of the extent of resection and detection of complications. The use of advanced MRI sequences could potentially provide additional physiological information that may aid in the preservation of healthy brain regions. This review aims to determine the added value of advanced imaging in ioMRI for pediatric brain tumor surgery compared to conventional imaging.

**Methods:** Our systematic literature search identified relevant articles on PubMed using keywords associated with pediatrics, ioMRI, and brain tumors. The literature search was extended using the snowball technique to gather more information on advanced MRI techniques, their technical background, their use in adult ioMRI, and their use in routine pediatric brain tumor care.

**Results:** The available literature was sparse and demonstrated that advanced sequences were used to reconstruct fibers to prevent damage to important structures, provide information on relative cerebral blood flow or abnormal metabolites, or to indicate the onset of hemorrhage or ischemic infarcts. The explorative literature search revealed developments within each advanced MRI field, such as multi-shell diffusion MRI, arterial spin labeling, and amide-proton transfer-weighted imaging, that have been studied in adult ioMRI but have not yet been applied in pediatrics. These techniques could have the potential to provide more accurate fiber tractography, information on intraoperative cerebral perfusion, and to match gadolinium-based T1w images without using a contrast agent.

**Conclusion:** The potential added value of advanced MRI in the intraoperative setting for pediatric brain tumors is to prevent damage to important structures, to provide additional physiological or metabolic information, or to indicate the onset of postoperative changes. Current developments within various advanced ioMRI sequences are promising with regard to providing in-depth tissue information.

## 1 Introduction

Pediatric brain tumor surgery aims for a complete resection of tumor tissue while avoiding damage to healthy functional brain regions. The extent of resection (EOR) is a key indicator of the child’s prognosis after surgery (Lindner et al., 2017; Tejada et al., 2018). An increased EOR could improve progression-free and overall survival (Marongiu et al., 2016; Costabile et al., 2019; Li et al., 2021) and reduce the risk of early reoperation (Shah et al., 2012; Sunderland et al., 2021).

The implementation of intraoperative magnetic resonance imaging (ioMRI) aims to sustain these goals by enabling “real-time” images of the brain, allowing for intraoperative evaluation of the extent of resection (EOR) (Marongiu et al., 2016). In 38% of pediatric ioMRI-guided surgical cases, the ioMRI was followed by additional resection leading to a substantial increase in EOR (Karsy et al., 2019; Giussani et al., 2022).

IoMRI may also contribute to alleviate a second challenge during surgery; avoiding damage to healthy functional brain regions and preserving the quality of life (Sunderland et al., 2021). Particularly, ioMRI can pinpoint intraoperative complications such as intracranial hemorrhage or tissue ischemia (Marongiu et al., 2016). IoMRI also gives a “real-time” update on the actual anatomy that may be affected by per-operative brain shift. All in all, ioMRI imaging may be used to update the neuronavigation that supports the neurosurgeon to achieve more radical tumor resections while avoiding neurological damage in surrounding brain tissue (Choudhri et al., 2015; Metwali et al., 2020; Sunderland et al., 2021).

Generally, ioMRI sites incorporate multiparametric imaging optimized for surgical aims. Conventionally used sequences in the pediatric ioMRI context are variations of 2D or 3D T1-and T2-weighted (T1w and T2w) images to visualize residual tumor tissue and to guide continuation of the neurosurgical resection (Abernethy et al., 2012; Choudhri et al., 2014; Millward et al., 2015; Giordano et al., 2017; Low et al., 2018; Tejada et al., 2018; Karsy et al., 2019).

Advanced MRI sequences, on the other hand, could potentially provide additional information on physiological aspects of the brain. For example, these sequences could assess the functional integrity of white matter tracts and blood perfusion or metabolic status of the brain tissue (Abernethy et al., 2012; Sanvito et al., 2021; Petr et al., 2022).

Potentially this might contribute to peroperative awareness and support prevention of damage to healthy functional brain regions.

In this manuscript, we systematically review the literature, aiming to answer the following question: “what is the added value of advanced imaging in ioMRI for pediatric brain tumor surgery, as compared to conventional imaging?”

## 2 Methods

We conducted a Pubmed search of the literature on advanced MRI in the pediatric ioMRI setting, based on the following search terms: (pediatr* OR paediatr* OR child*) AND (ioMRI OR iMRI OR iopMRI OR “intraoperative MRI” OR “intra-operative MRI”) AND (tumor* OR tumour* OR glioma*). Papers were screened on title and abstract, and relevant articles were read in full text.

Articles were included based on the following criteria:

- Case series, cohorts, or trials including pediatric patients (age <19 years) undergoing ioMRI for brain (tumor) surgery.
- The reported MRI sequences were intraoperatively acquired.
- The added value of advanced MRI sequences was reported.

Literature reviews, book chapters, articles on epilepsy, *in vitro* studies, articles not written in English, or with no focus on surgery; articles on low-field MRI (<1.5 Tesla), no focus on ioMRI or no pediatric patients (age, <19 years) were excluded.

Conventional MRI sequences were defined as T1w, T2w, or fluid-attenuated inversion recovery (FLAIR) sequences (with or without gadolinium contrast) that can provide structural information about the brain (Lequin and Hendrikse, 2017). Advanced MRI sequences were defined as sequences that could also provide information on physiology and functionality, including metabolism, brain tumor cellularity, and hemodynamics (Lequin and Hendrikse, 2017; Petr et al., 2022).

The following MRI sequences were considered as advanced MRI sequences: arterial spin labelling (ASL), amide-proton transfer-weighted imaging (APTw), dynamic contrast-enhanced (DCE), dynamic susceptibility contrast (DSC), diffusion kurtosis imaging (DKI), intravoxel incoherent motion (IVIM), multicomponent-driven equilibrium single pulse observation of T1 and T2 (mcDESPOT), magnetic resonance spectroscopy (MRS), myelin water imaging (MWI), neurite orientation and dispersion density imaging (NODDI), quantitative magnetization transfer (qMT), quantitative susceptibility mapping (QSM), relaxometry, vascular, extracellular and restricted diffusion for cytometry in tumors (VERDICT) (Petr et al., 2022); diffusion-weighted imaging (DWI), diffusion tensor imaging (DTI), chemical shift imaging (CSI), susceptibility-weighted imaging (SWI), and functional MRI (Lequin and Hendrikse, 2017).

From each included study, the following data were extracted: study center, magnetic field strength (Tesla), number of brain tumor patients included, histopathological diagnosis, advanced MRI sequences used, and their added value as reported in the study.

As this search yielded limited information, we extended our search beyond the initial research question, using the snowball technique, to gain more information on the technical background of each advanced MRI technique, their use in ioMRI in adults, and their use in routine pediatric brain tumor care.

## 3 Results

### 3.1 Systematic literature review

**FIGURE 1:**
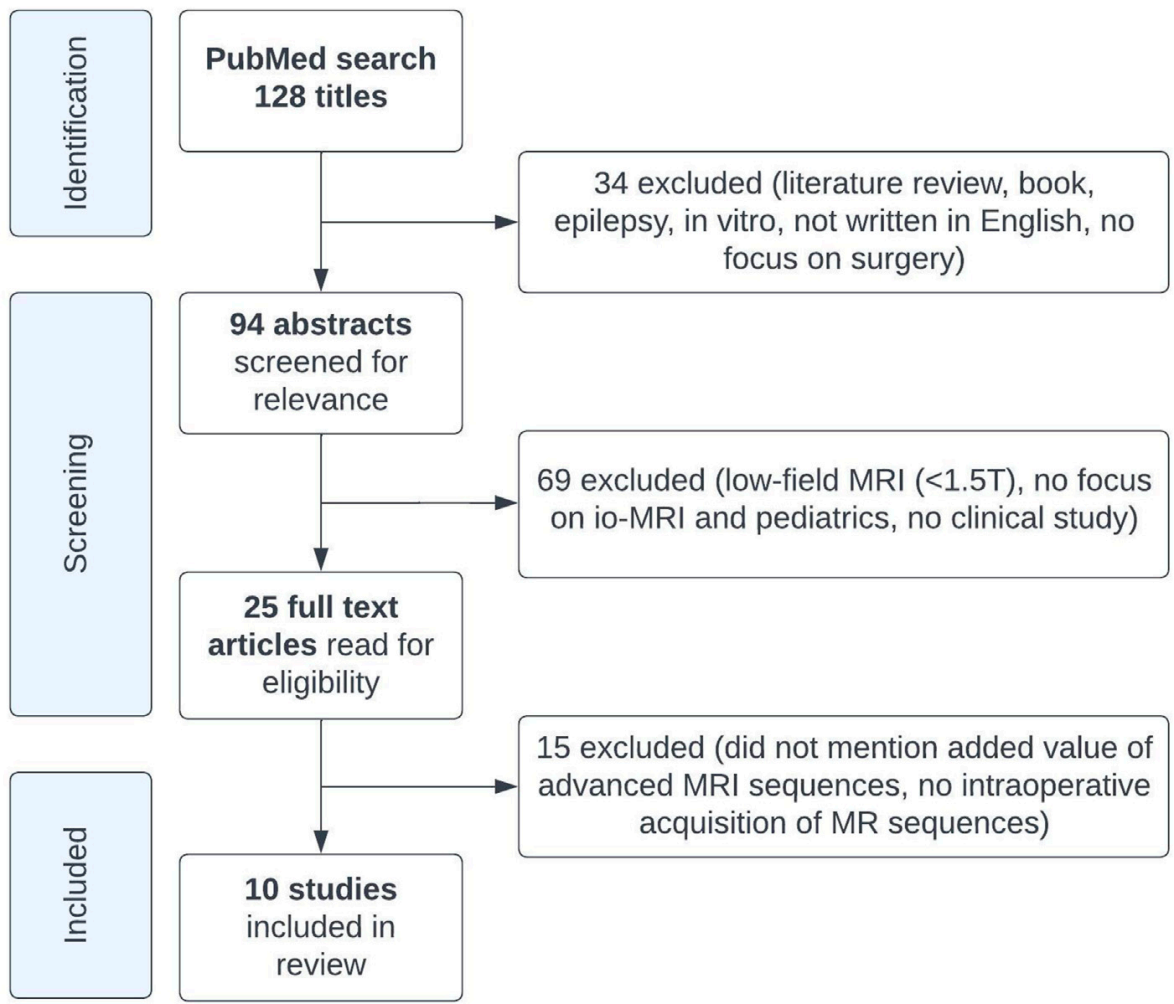
Study flowchart.

The literature search on PubMed yielded 128 articles, of which ten met our inclusion criteria (Figure 1; Table 1) (Abernethy et al., 2012; Yousaf et al., 2012; Avula et al., 2013; Ren et al., 2013; Giordano et al., 2017; Tejada et al., 2018; Saint-Martin et al., 2019; Low et al., 2020; Avula et al., 2021; Sunderland et al., 2021). Most of these studies were cohort studies, two were case series (Abernethy et al., 2012; Ren et al., 2013), one included a review of experience (Abernethy et al., 2012), and one included a comparison of their results to existing literature (Low et al., 2020). A total of 604 ioMRI brain tumor patients were described in the included literature. All studies that resulted from our PubMed search were performed on either a 1.5 or 3 Tesla MRI scanner. These studies aimed to report the initial ioMRI experience, to evaluate early repeat resection (Avula et al., 2013), to detect ischemic infarcts on diffusion ioMRI (Saint-Martin et al., 2019), or to evaluate ioMRI scans as post-operative scans (Avula et al., 2021). The included studies described a heterogeneous group of histopathological brain tumors with advanced MRI (Table 1). Just one study focused specifically on advanced ioMRI (Saint-Martin et al., 2019) but did not perform any post-processing for further data analysis. Eight studies were evaluated as having a low risk of bias as they included consecutive patients of all histopathological brain tumors though five of these studies reported advanced imaging only in selective patients (Abernethy et al., 2012; Yousaf et al., 2012; Avula et al., 2013; Tejada et al., 2018; Low et al., 2020), introducing an increased bias risk. Two studies had a higher risk of bias as they selected based on tumor type; subendymal giant cell astrocytoma (Ren et al., 2013) or thalamic tumor patients (Sunderland et al., 2021).

**TABLE 1:** Study designs and parameters of included studies.

Study	Included pediatric ioMRI brain tumor patients [total]	Study design	Study aim	Brain tumor types described with advanced MRI (histopathology or location)	Advanced MRI sequence	Added value
Abernethy et al. (2012)	Not mentioned	Review of experience and case series	Report of initial ioMRI experience	Not mentioned	DTI	Reconstruction of fibers to avoid damage to important structures.
Alder Hey Children’s Hospital, Liverpool, United Kingdom					DSC	Early resection control and information on relative cerebral blood flow.
Yousaf et al. (2012)	73 [73]	Cohort	Report of initial ioMRI experience	Deep-seated tumors (DTI), pathology not provided	DTI	Reconstruction of fibers to avoid damage to important structures.
Alder Hey Children’s Hospital, Liverpool, United Kingdom					DSC	Early resection control and information on relative cerebral blood flow.
					MRS	Evaluation of presence of abnormal metabolites.
Avula et al. (2013)	36 [72]	Cohort	Evaluation of early repeat resection	Atypical teratoid rhabdoid tumor (MRS)	DWI	Detect diffusion restriction that can indicate hemorrhage or ischemic infarcts.
Alder Hey Children’s Hospital, Liverpool, United Kingdom					DSC	Information on relative cerebral blood flow.
					MRS	Evaluation of presence of abnormal metabolites.
Ren et al. (2013)	7 [7]	Case series	Report of initial ioMRI experience in SEGA patients	SEGA	DTI	Reconstruction of corticospinal tract and arcuate fasciculus to avoid damage to important structures.
PLA General Hospital, Beijing, China						
Giordano et al. (2017)	75 [75]	Cohort	Report of ioMRI experience	Craniopharyngioma, Rathke’s cleft cyst, pituitary macroadenoma, pilocytic astrocytoma, diffuse astrocytoma, glioblastoma, oligodendroglioma, angiocentric glioma, SEGA, anaplastic astrocytoma, ependymoma, cortical dysplasia, ganglioglioma, hamartoma, germinoma, PNET.	DTI	Reconstruction of fibers to avoid damage to important structures.
International Neuroscience Institute–Hannover, Hannover, Germany						
Tejada et al. (2018)	223 [223]	Cohort	Report of ioMRI experience	High-grade glioma (DTI, CSI), midline glioma, and multifocal embryonal tumor (DWI).	DWI	Detect diffusion restriction that can indicate hemorrhage or ischemic infarcts.
					DTI	Reconstruction of fibers to avoid damage to important structures.
Alder Hey Children’s Hospital, Liverpool, United Kingdom					DSC	Information on relative cerebral blood flow.
					MRS and CSI	Evaluation of presence of abnormal metabolites that could either represent edema or tumor invasion.
Saint-Martin et al. (2019)	115 [115]	Cohort	Detection of ischemic infarct on diffusion ioMRI	Medulloblastoma, pilocytic astrocytoma, glioblastoma, anaplastic ependymoma, craniopharyngioma, epidermoid cyst, anaplastic ganglioglioma, desmoplastic infantile ganglioglioma, and hypothalamic hamartoma.	DTI	Generate B0 and ADC maps to visualize ischemic infarcts.
The Montreal Children’s Hospital, Montreal, Canada						
Low et al. (2020)	35 [43]	Cohort and comparison to literature	Report of ioMRI experience	Not mentioned	DTI	Reconstruction of corticospinal tract to avoid damage to important structures.
KK Women’s and Children’s Hospital, Singapore, Singapore						
Avula et al. (2021)	20 [20]	Cohort	Evaluation of ioMRI scan as post-operative scan	Medulloblastoma, pilocytic astrocytoma, fibrillary astrocytoma, ganglioglioma, craniopharyngioma, high-grade glioma, pleomorphic xanthoastrocytoma, pilomyxoid astrocytoma, SEGA, and pituitary adenoma.	DWI and DTI	Detect diffusion restriction that can indicate hemorrhage or ischemic infarcts.
Alder Hey Children’s Hospital, Liverpool, United Kingdom						
Sunderland et al. (2021)	20 [30]	Cohort	Report of ioMRI experience in thalamic tumor patients	Thalamic tumors	DTI	Reconstruction of fibers to avoid damage to important structures.
Alder Hey Children’s Hospital, Liverpool, United Kingdom						

IoMRI = intraoperative MRI; DTI = diffusion tensor imaging; DSC = dynamic susceptibility contrast; MRS = magnetic spectroscopy resonance; SEGA = subependymal giant cell astrocytoma; PNET = primitive neuroectodermal tumor.

The ten articles that reported the use of advanced ioMRI sequences for pediatric brain tumor surgery focused on diffusion MRI (DWI and DTI), perfusion MRI (DSC), and metabolic MRI (MRS and CSI). Diffusion MRI was used in 288 patients, covered by seven studies (Ren et al., 2013; Giordano et al., 2017; Tejada et al., 2018; Saint-Martin et al., 2019; Low et al., 2020; Avula et al., 2021; Sunderland et al., 2021). Three other studies that covered diffusion MRI did not specify the number of patients. Most authors used DWI to detect diffusion restriction that can indicate hemorrhage or ischemic infarcts and DTI for reconstructing fibers (i.e., corticospinal tract and arcuate fasciculus) to avoid damage to important structures. DTI was also used to generate B0 and apparent diffusion coefficient (ADC) maps to visualize ischemic infarcts (Saint-Martin et al., 2019). Perfusion MRI was used in 22 patients, covered by one study (Tejada et al., 2018). Three other studies also used perfusion MRI for selective patients but did not specify their numbers. Authors used DSC for early resection control and additional physiological information on relative cerebral blood flow. Metabolic MRI was used in 11 patients, covered by one study (Tejada et al., 2018). Two other studies that reported use of MRS did not specify the number of selected patients. MRS and CSI were used to evaluate the presence of abnormal high concentration of metabolites (i.e., choline) that could either represent edema or tumor invasion.

In conclusion, the sparse literature demonstrated that advanced sequences in ioMRI for pediatric brain tumor surgery was used to reconstruct fibers to prevent damage to important structures, provide information on relative cerebral blood flow or abnormal metabolites, or to indicate the onset of hemorrhage or ischemic infarcts.

### 3.2 Explorative literature review

The explorative literature search was confined to the same advanced MRI fields: diffusion-, perfusion-, and metabolic MRI. It focused on the technical background of the sequences, their use in the ioMRI setting for adult brain tumor surgery, and their use in routine pediatric brain tumor care.

### 3.3 Diffusion MRI

#### 3.3.1 Technical background of diffusion MRI

Diffusion MRI (dMRI) is based on the diffusion of water molecules and provides information on the microstructural tissue organization. In dMRI, multiple diffusion-weighted images are acquired in multiple spatial directions.

Diffusion tensor imaging (DTI) leverages dMRI data acquired at a single diffusion weighting (b-value) that is therefore called single-shell acquisition (Table 2). It is the conventional dMRI quantification method in clinical practice. The acquisition of dMRI data with multiple b-values, called multi-shell, has recently become feasible within clinically acceptable acquisition times. Multi-shell dMRI allows for more advanced quantification models like diffusion kurtosis imaging (DKI).

**TABLE 2:** Difference between single- and multi-shell diffusion MRI acquisition for brain tumor imaging.

	Single-shell diffusion MRI	Multi-shell diffusion MRI
Typical quantification methods	DTI	DKI
Typical b-values	Single, around b = 1000 s/mm^2^.	Multiple, between b = 1,000 and b = 3,000 s/mm^2^. For example, b = 1,000, 2000, 3,000 s/mm^2^.
Fiber tractography model	Depending on the number of gradient directions: • <28, DTI. Unable to resolve crossing fibers. • ≥28, spherical deconvolution. Can (partly) resolve crossing fibers^*^	Advanced methods applicable if at least 28 gradient directions (45+ recommended) are collected for the largest b-value. Can resolve crossing- and kissing fibers, and account for partial volume effects (e.g., fluids).

DTI = diffusion tensor imaging; DKI = diffusion kurtosis imaging; b-value = Diffusion weighting.

* Guo et al. (2020).

**FIGURE 2:**
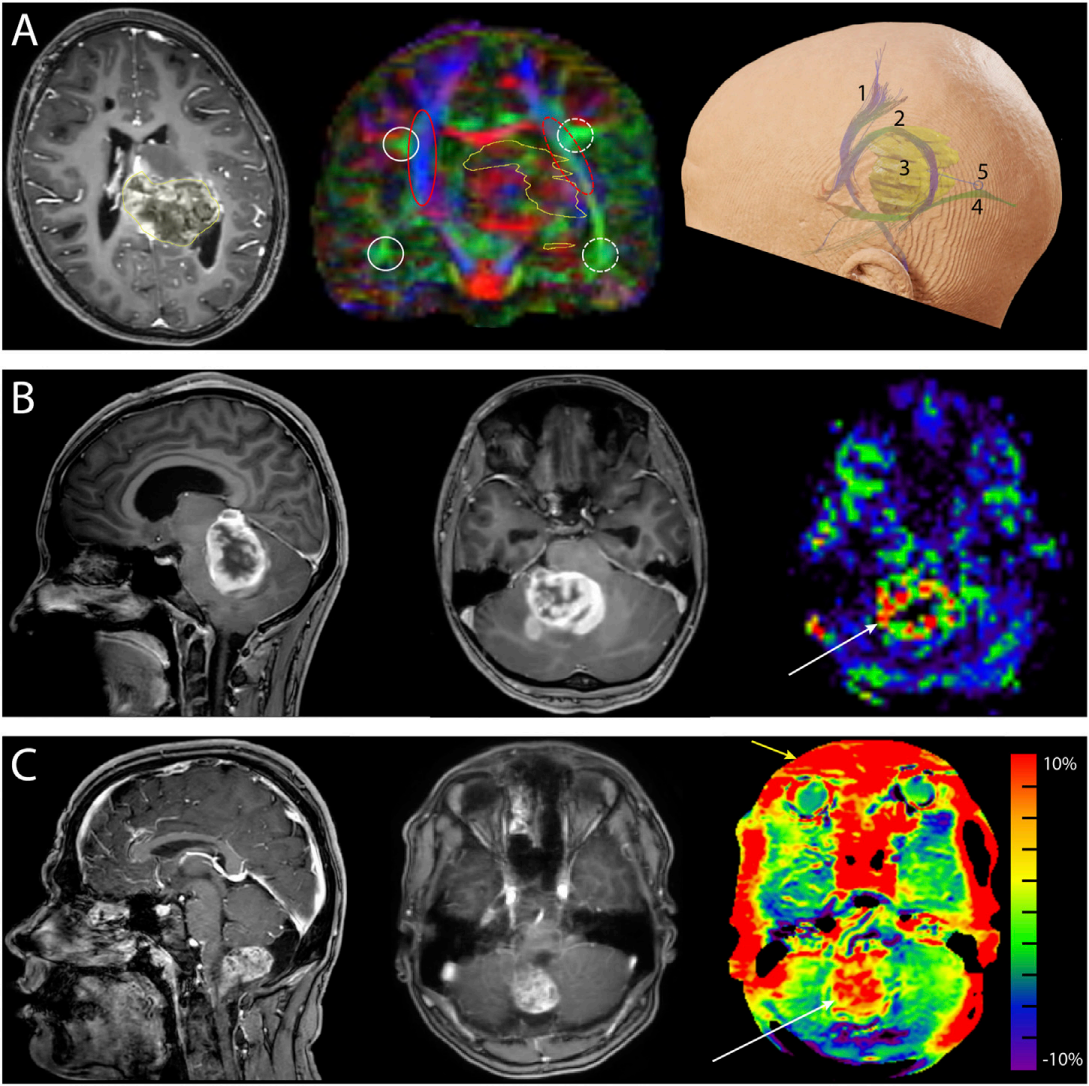
Neurosurgical cases demonstrating the added value of advanced MRI. **(A)** Preoperative images of a 10-year-old girl with a diffuse midline glioma (H3K27 mt) originating from the left posterior thalamus and mesencephalon and expanding into the atrium of the left ventricle. *Left*: the transverse T1-weighted contrast-enhanced image shows enhancement of the tumor (yellow outline). *Center:* the coronal fractional anisotropy color-coded map (single-shell diffusion MRI, 16 directions) shows left-right asymmetry demonstrating the displacement of fibers caused by the tumor (yellow outline). The white circles depict the arcuate fasciculus, and the red ovals depict the corticospinal tracts. Solid lines depict the unaffected side, and the dashed lines show the affected side. *Right:* reconstruction of the corticospinal tract 1), arcuate fasciculus 2), tumor 3), and optic radiation 4). A parietal surgical approach posterior to the arcuate fasciculus and superior to the optic radiation 5) was chosen for tumor mass reduction and histopathological diagnosis. **(B)** Preoperative images of a 17-year-old girl with neurofibromatosis type 1 and a space-occupying lesion in the fourth ventricle. *Left*: the sagittal T1-weighted contrast-enhanced images. The differential diagnosis was pilocytic astrocytoma or high-grade glioma. *Center*: transverse T1-weighted contrast-enhanced image. *Right*: the hyperperfusion (white arrow) of the unquantified arterial spin labeling image makes diagnosing a high-grade glioma more probable. Histopathological examination revealed a high-grade glioma with pilocytic features. **(C)** Preoperative images of a 17-year-old boy with a pilocytic astrocytoma. *Left:* sagittal T1-weighted contrast-enhanced image. *Center:* transverse T1-weighted contrast-enhanced image. *Right:* amide-proton transfer-weighted (APTw) image. Note the hyperintense region (white arrow) that matches the contrast enhancement on T1-weighted contrast-enhanced image. The red outer rim (yellow arrow) of the APTw image is likely caused by susceptibility-weighted air-tissue artifacts. Ethical approval from the local medical ethics committee was obtained for this study.

DMRI metrics such as the apparent diffusion coefficient (ADC) and fractional anisotropy (FA) can be used to disentangle tissue components (e.g., cellular mass versus edema or other cavities) that can be useful for clinical decision-making (Lequin and Hendrikse, 2017). DKI has been shown to be more sensitive to microstructural changes than DTI (Mohammadi et al., 2015; Yeh et al., 2021). Moreover, multi-shell dMRI data allowed models that could capture the presence of multiple water components (Rydhög et al., 2017), such as free water (e.g., edema) (Pasternak et al., 2009) or perfusion (e.g., intra-voxel incoherent motion) (De Luca et al., 2017). In addition to microstructural properties, dMRI data can be used to reconstruct the trajectory of brain white matter pathways (Figure 2A) (Jeurissen et al., 2019). When such fiber tractography models are based on multi-shell dMRI, they could typically account for crossing fibers and properties of different tissue types in the brain (e.g., white matter versus grey matter versus free fluid). Multi-shell data could be used to generate a more detailed and anatomically accurate fiber tractography than single-shell dMRI (Table 4) (Poretti et al., 2012; Mohammadi et al., 2015; Guo et al., 2020; Yeh et al., 2021). Moreover, it might be able to map the border of major white matter tracts and displaced fiber tracts more reliably (Nimsky, 2014; De Luca et al., 2020). Disadvantages of multi-shell dMRI are lower signal-to-noise ratio (SNR), which is often resolved with a lower spatial resolution or longer acquisition time, and technically demanding post-processing to increase image quality (Mohammadi et al., 2015). Both single- and multi-shell dMRI are sensitive to eddy currents and susceptibility artifacts between air and tissue (Table 3). These artifacts are increased in the intraoperative setting due to the open skull (Mohammadi et al., 2015; Lindner et al., 2022). However, this might be worse in multi-shell diffusion MRI acquisition due to the higher gradient amplitudes (Mohammadi et al., 2015).

**TABLE 3:** Technical considerations and details of each advanced MRI sequence.

Sequence	Estimated average scan time^*^	Desired resolution^§^	Sequence-specific artifacts and considerations
Single-shell dMRI	3 min (in case of DTI, whole-brain, about 20 directions)^†^	Isotropic, <2.5 mm	Susceptible to air-tissue artifacts and eddy currents; Susceptible to artifacts caused by free diffusion in tissue (e.g., edema).
Multi-shell dMRI	8 min (in case of a minimum protocol for DKI, whole-brain, about 42 directions)^‡^	Isotropic, <2.5 mm	Susceptible to air-tissue artifacts and eddy currents; Higher b-value acquisition results in a lower SNR that needs to be compensated by averaging or reducing the echo time; Longer acquisition time.
ASL	4 min 30 s (whole-brain)	3 × 3 × 7 mm	Susceptible to air-tissue artifacts and blood flow artifacts caused by pulsation of large blood vessels; Thick slices (7 mm) required to reach desired SNR levels in the clinical setting; Difficult to position ASL labeling box perpendicular to vessels in the neck when the patient has a twisted neck in the surgical position.
MRS (single-voxel)^||^	4 min 28 s	20 × 20 × 20 mm	Susceptible to air-tissue artifacts; Long acquisition time and low spatial resolution.
APTw imaging	4 min (half-brain)	0.9 × 0.9 × 6 mm	Susceptible to air-tissue artifacts causing a B0 offset of the saturation pulse^¶^, thereby less specific for APTw signal; Thick slices (6 mm) required to reach desired SNR level in the clinical setting.

DMRI = diffusion MRI; DTI = diffusion tensor imaging; DKI = diffusion kurtosis imaging; SNR = signal-to-noise ratio; ASL = arterial spin labelling; MRS = magnetic resonance spectroscopy; APTw = Amide proton transfer-weighted.

*Estimated scan time when making use of accelerated imaging techniques on a 3 Tesla strength while using two single-loop receiving coils.

^†^Whole brain is a field of view of 240 × 240 × 160 mm.

^‡^The acquisition of multi-shell dMRI with single-loop receiving coils cannot be accelerated with the multi-band technique as it is only available for a conventional multi-channel head coil.

^§^Reconstructed voxel size.

^||^Semi-LASER spectroscopy with an echo time of 35 ms.

^¶^ Zhou et al. (2019).

#### 3.3.2 Use of diffusion MRI in adult ioMRI setting

In the adult ioMRI setting, dMRI was used for fiber tracking of white matter fibers in eloquent brain areas (Zhang et al., 2022). Due to the brain shift after craniotomy, such fiber reconstructions had to be adjusted intraoperatively (Zhang et al., 2022). Single-shell dMRI, which is suitable for DTI, has been used more often in clinical practice than multi-shell dMRI due to its reliance on simpler data acquisition and reconstruction models (Mohammadi et al., 2015). Previous research on intraoperative single-shell dMRI reported its use in the estimation of brain shift (Metwali et al., 2020). It was also an integral part of an ioMRI protocol which was able to increase EOR from 44% to 88.5% in an adult glioblastoma population (Marongiu et al., 2016). Studies comparing EOR in groups of patients using ioMRI protocols with and without diffusion MRI are not currently available and would be helpful to determine its specific added value. Hypothetically, multi-shell dMRI might even further improve these findings (Nimsky, 2014). The feasibility of multi-shell dMRI fiber tractography was reported by Leote et al. (2018) for the pre-surgical planning of adult brain tumor surgery.

#### 3.3.3 Use of diffusion MRI in pediatric routine brain tumor care

DMRI metrics, such as the ADC and FA, were used in routine pediatric clinical practice to grade tumor tissue and differentiate it from healthy brain tissue (Table 4) (Ouadih et al., 2022). ADC was associated with cellularity in previous studies, which was correlated with extracellular diffusion (Villanueva-Meyer et al., 2017; She et al., 2021). Reduced diffusivity (low ADC) compared to surrounding tissues could point toward high cellular tumor tissue (i.e., medulloblastoma) with little extracellular water and ischemia. Conversely, high diffusivity (high ADC) could indicate increased extracellular water, vasogenic edema, or necrotic tissue (Avula et al., 2014). FA values were also used to grade brain tumor tissue in children, where low FA has been associated with high-grade glioma (Poretti et al., 2012). Furthermore, DKI metrics were considered more promising than conventional dMRI metrics in tumor grading and prediction of the expression of Ki-67, a histopathological cell proliferation biomarker (Jiang et al., 2015; Sanvito et al., 2021).

**TABLE 4:** Clinical implications of advanced ioMRI in pediatrics.

Imaging technique	Promises	Pitfalls
Single-shell dMRI	Conventional quantification method in clinical practice; DTI metrics (e.g., MD and FA) are reliable to grade tumor tissue.	Data is likely not optimal for fiber tractography in presence of edema and/or fluid cavities.
Multi-shell dMRI	Sensitive to additional effects than single-shell dMRI (e.g., diffusion restrictions due to membranes). Suitable for state-of-the-art fiber tractography methods to resolve crossing- and kissing fibers and account for partial volume effects; DKI metrics can be used to grade tumor tissue and predict Ki-67 expression; Detect residual diffusion restriction effects and residual tumor tissue.	Not commonly used in clinical practice; Initially requires more expert knowledge to set-up. Lack of user-friendly tools to leverage its full potential.
ASL	Detect residual tumor tissue; Differentiate from non-tumorous gadolinium enhancement in resection cavity; Mapping of functional areas.	Artifacts in the control image can propagate in ASL difference image (e.g., false-positive hyperperfusion).
MRS (single-voxel)	Metabolic evaluation of tumor tissue; Metabolite pattern recognition could be assisted by automated processing.	Low specificity of tumor type.
APTw imaging	Replace gadolinium-based anatomical sequences to detect residual tumor; Can generate a quantified image; APTw signal could be associated with increased protein levels.	Not commonly used in clinical practice; Potentially low detection sensitivity to low-grade gliomas which are more prominent in pediatrics.

dMRI = diffusion MRI; DTI = diffusion tensor imaging; DKI = diffusion kurtosis imaging; MD = mean diffusivity; FA = fractional anisotropy; ASL = arterial spin labeling; MRS = magnetic resonance spectroscopy; APTw = amide-proton transfer-weighted.

### 3.4 Perfusion MRI

#### 3.4.1 Technical background of contrast-based perfusion MRI

Brain tumor perfusion characteristics can be investigated with gadolinium contrast-based perfusion MRI sequences. Examples of such imaging methods are dynamic susceptibility contrast- (DSC) and dynamic contrast-enhanced (DCE) imaging (Lequin and Hendrikse, 2017). Gadolinium contrast is the cornerstone of MRI tumor diagnostics, but its disadvantages should be considered carefully. First, gadolinium could cause the accumulation of toxic side products, especially in renal failure patients, increasing the chance of developing nephrogenic systemic fibrosis (Sadowski et al., 2007). Second, gadolinium is a blood-pool contrast agent whose enhancement assumes an intact blood-brain barrier. However, surgical manipulation violates this assumption in the intraoperative setting. This could lead to misinterpretation of gadolinium contrast enhancement (Abernethy et al., 2012). Third, gadolinium could cause a delay in sequence repetition if readministered within 24 h. However, on the day of the surgical procedure, MR scan sessions are often repeated (e.g., intra- and post-operative MRI or multiple ioMRI sessions) (Millward et al., 2015; Keil et al., 2018). Fourth, gadolinium may pose an environmental threat (Trapasso et al., 2021). Taking these disadvantages of gadolinium contrast-based sequences into account, we shall focus on non-invasive alternatives for this explorative literature search on perfusion MRI.

#### 3.4.2 Technical background of non-invasive perfusion MRI

Arterial spin labelling (ASL) is a perfusion MRI sequence that can quantify absolute CBF based on endogenous blood water (Keil et al., 2018). The ASL signal is based on subtracting two consecutive images (Lindner et al., 2022). The first image labels inflowing arterial blood at the cervical level that is magnetically inverted with a radiofrequency pulse (Alsaedi et al., 2018; Keil et al., 2018). This image is acquired after an appropriate delay time, called the post-labeling delay, which depends on the speed of blood flow and thus the health of the vascular tree (Carsin-Vu et al., 2018; Keil et al., 2018). The second image is the control image that covers the same downstream cerebral region of interest but without magnetically inverting the blood in the cervical arteries (Alsaedi et al., 2018). The ASL difference image visualizes the perfusion signal from the arteries into neighboring brain tissue (Alsaedi et al., 2018). This difference image can then generate a map that represents the quantified CBF in mL/100 g brain tissue/min (Keil et al., 2018).

ASL has the advantage that it is not susceptible to blood-brain barrier leakage artifacts usually observed for gadolinium (Lindner et al., 2017; Keil et al., 2018). Another important advantage is that it can be easily repeated without any cost, except for adding the scanning duration. Lastly, the ability of ASL to quantify absolute CBF (Keil et al., 2018) is useful for assessment of cerebral vitality in surgical and eloquent areas. Disadvantages of ASL are the relatively low SNR (Table 3) and limited sensitivity in low CBF regions such as white matter (Petr et al., 2022). However, the low SNR of the ASL signal is less prominent in children (Yeom et al., 2013). The problem of limited sensitivity in the white matter could be overcome by integrating ASL and dMRI in a multiparametric model to generate a comprehensive clinical overview.

#### 3.4.3 Use of non-invasive perfusion MRI in adult ioMRI setting

Intraoperative ASL could be used for iatrogenic changes in CBF and to depict residual tumor tissue in adults (Table 4) (Lindner et al., 2017). Lindner et al. (2017) reported the feasibility of intraoperative residual tumor detection using ASL when compared to gadolinium-contrast based T1w images (T1w-Gd) in adults with glioblastoma. They argued that ASL could make a more definite judgment of residual tumor tissue than conventional ioMRI sequences (Lindner et al., 2017). However, only a small number of patients were included in this study (n = 8). Another use of intraoperative ASL could be mapping functional areas that should not be damaged during surgery to avoid postsurgical deficits (Lindner et al., 2022). This research also focused on an adult glioblastoma population. It showed that intraoperative ASL could reliably map functional areas and residual brain tumor after post-processing special data. However, their analysis method also revealed false-positive artifacts on the resection rim that should be carefully considered.

#### 3.4.4 Use of non-invasive perfusion MRI in pediatric routine brain tumor care

Several studies have indicated that ASL could be a reliable method to evaluate perfusion patterns of brain tumors in pediatric populations (Yeom et al., 2013; Morana et al., 2017). ASL-based CBF maps have been used routinely to grade tumor tissue in children due to its correlation with tumor vascular density (Yeom et al., 2013; Dangouloff-Ros et al., 2016; Keil et al., 2018). In such a way, hyperperfusion on ASL images could indicate the high malignity of tumor tissue, because of increased tumor tissue activity and blood flow supply (Figure 2B) (Yeom et al., 2013; Dangouloff-Ros et al., 2016; Keil et al., 2018). To illustrate this, Yeom et al. (2013) presented a case diagnosed with mixed anaplastic astrocytoma-glioblastoma that showed elevated CBF values in tumor tissue compared to non-tumoral grey matter. On the other hand, low-grade gliomas, such as dysembryoplastic neuroepithelial tumors, showed low ASL-based CBF within the tumor region (Yeom et al., 2013; Lequin and Hendrikse, 2017).

Some limitations of ASL in pediatric routine brain tumor care should be considered. First, various ASL perfusion patterns existed for both high- and low-grade gliomas depending on vascular characteristics (e.g., vessel density and capillary exchange rate). Second, ASL hypoperfusion in children could also be caused by edema or scar tissue (Keil et al., 2018). Due to this heterogeneity of measurements in different tumor types, ASL images should be considered in combination with high-resolution anatomical images for a more definite judgement. Third, the effect of anesthesia on regional CBF should also be considered, as general anesthesia usually induces vasodilation (Carsin-Vu et al., 2018; Keil et al., 2018).

### 3.5 Metabolic MRI

#### 3.5.1 Technical background in metabolic MRI

Proton magnetic resonance spectroscopy (MRS) is a non-invasive metabolic MRI technique that can detect metabolites in the tissues (Wilson et al., 2019). For example, MRS could detect the neurotransmitters glutamate, glutamine, GABA, and other metabolites such as N-acetyl aspartate, choline, creatine, and myo-inositol. Alterations in metabolite levels could give insight into the pathophysiological condition of tissue (Petr et al., 2022). A limitation of MRS is that on clinical field strengths (3 Tesla), the sensitivity is relatively low as usually, the scan time in the clinic is limited (Table 3) (Wilson et al., 2019). Therefore large voxel sizes of 8 mL are commonly used for MRS in clinical practice (Wilson et al., 2019). This could be problematic in the intraoperative setting where whole-brain images are preferred to evaluate spatial heterogeneity. In that setting, the acquisition time for whole-brain chemical shift-imaging (CSI) (or even a single slice) becomes a practical limitation. Currently, fast whole brain CSI methods are not implemented as vendor products for use in intraoperative setting.

Another metabolic MRI modality is chemical exchange saturation transfer (CEST). CEST is an MRI modality that exploits the abundance of exchangeable protons of a certain metabolite, and its chemical exchange with water protons, to image the relative concentration of a certain metabolite (Wu et al., 2016). In CEST, protons of the metabolite of interest are saturated by a prolonged saturation RF pre-pulse; during this pre-pulse, water exchanges unsaturated protons with saturated protons from the metabolites of interest, resulting in a reduction of the water signal, which can be imaged over the whole volume. This has the advantage of an easier interpretation in clinical practice compared to single-voxel MRS (Wu et al., 2016). Amide-proton transfer-weighted (APTw) imaging is a form of CEST imaging sensitive to chemical exchange of protons in water, mobile proteins, and peptides. This form of CEST has been mainly used for brain tumors (Wu et al., 2016; Suh et al., 2019).

#### 3.5.2 Use of metabolic MRI in adult ioMRI setting

In the intraoperative setting, metabolic MRI could be used to provide biochemical information about relative metabolite concentrations of potential residual tumor tissue (Pamir et al., 2010; Yousaf et al., 2012). Pamir et al. (2010) reported that the combination of MRS with DWI effectively differentiated peritumoral changes from a residual tumor in adult low-grade glioma. APTw imaging has not yet been described in the intraoperative setting. However, APTw imaging has been reported to guide stereotactic biopsy in adults with newly diagnosed gliomas (Jiang et al., 2017). Jiang et al. (2017) showed that the APTw signal was sensitive and specific for differentiating between adult low- and high-grade gliomas.

Hypothetically, intraoperative APTw imaging might be an alternative to a T1w-Gd sequence (Figure 2C). Yu et al. (2019) reported that lesions identified on the APTw images mimicked those on the T1w-Gd images of adult meningioma patients. To add to this argument, APTw images could provide improved diagnostic specificity compared to T1w-Gd images in high-grade glioma patients (Zhou et al., 2013). APTw imaging accurately differentiated between glioblastoma and solitary brain metastases in adults (Yu et al., 2017). Also, Zhou et al. (2019) showed that APTw images added new information to the standard T1w-Gd image in an oligodendroglioma case. A disadvantage of intraoperative APTw images could be that surgery-induced blood components could produce hyperintensity artifacts on APTw images (Zhou et al., 2019; Zhang et al., 2021). Moreover, whether the thicker slices used for APTw images in the clinic still add valuable information in the surgical setting is questionable. Lastly, the open skull during surgery could increase magnetic field (B0) inhomogeneities due to the sensitivity of the APTw signal to air-tissue interfaces which decreases accuracy of the APTw signal (Zhou et al., 2019). To solve this issue, attention should be paid to remove all air bubbles in the brain and B0 shimming to avoid these susceptibility artifacts during intraoperative APTw image acquisition (Table 3). An alternative could be to focus B0 shimming and CEST acquisition on a specific region of interest instead of imaging the whole brain. Taking technical and logistical limitations into account, intraoperative APTw imaging seems to be the most useful in the case of high-grade glioma patients (Chalil and Ramaswamy, 2016; Zhang et al., 2021).

#### 3.5.3 Use of metabolic MRI in pediatric routine brain tumor care

MRS has been used in clinical practice to support diagnosing and differentiating brain tumor subtypes in children (Faghihi et al., 2017). In such a way, spectroscopic patterns can be distinctive for tumor subtypes (Vicente et al., 2013). Aggressive features of a tumor could be indicated by an elevated choline-to-N-acetyl aspartate ratio or the presence of lactate (Choudhri et al., 2015). The recognition of these patterns could be assisted by automated processing in pediatrics (Vicente et al., 2013). Due to regional metabolic variations, MRS alone did not suffice to define all regional components of tumors (Lequin and Hendrikse, 2017). MRS should be combined with other standardized MRI methods for a more definitive diagnosis. For example, based on the metabolic profile alone, a pilocytic astrocytoma could be misdiagnosed as a more aggressive variant due to a higher choline peak than a creatine peak and an elevated lipid peak (Table 4) (Lequin and Hendrikse, 2017).

APTw imaging could also be used in pediatric brain tumor care to grade and identify the proliferative activity of tumor tissue (Zhang et al., 2021). The increased protein levels in tumor tissue could be indicated by increased APTw values that might be positively correlated with Ki-67 expression levels (Table 4) (Wu et al., 2016; Suh et al., 2019). Therefore, high-grade gliomas could be indicated by a higher APTw signal than low-grade gliomas, although these results were more heterogeneous in the pediatric population (Suh et al., 2019; Zhang et al., 2021). Besides, APTw imaging was said to differentiate between brain tumor tissue and edema (Wen et al., 2010). Zhang et al. (2020) presented a pediatric case where the edema showed a similarly low APTw signal as the healthy surrounding tissue. However, a comparison of the APTw signal between tumor tissue and edema was missing. Due to the scarce existing literature in the pediatric population, further research is essential to reliably incorporate APTw imaging in clinical practice (Suh et al., 2019).

## 4 Discussion

This study is a retrospective study of the literature on the added value of advanced MRI in the intraoperative setting of pediatric brain tumors compared to conventional MR imaging. Our systematic literature search revealed that the fields of diffusion-, perfusion-, and metabolic MRI have been reported for selective cases during surgery. The available literature was sparse and demonstrated that advanced sequences were used to prevent damage to reconstruct fibers to prevent damage to important structures, provide information on relative cerebral blood flow or abnormal metabolites, or to indicate the onset of hemorrhage or ischemic infarcts.

Our explorative literature search revealed developments within each advanced MRI field that have been studied in the adult ioMRI population but have not yet been applied in pediatrics. First, multi-shell dMRI could offer “real-time” fiber tractography that was said to be more anatomically accurate than models based on single-shell data. Second, ASL could give information on intraoperative cerebral perfusion and could indicate residual tumor tissue intraoperatively without a contrast agent. Third, APTw imaging and ASL could potentially match T1w-Gd images. Despite these promising advances, the technical and practical limitations of each of these advanced MRI sequences should be carefully considered before implementing them in standard pediatric ioMRI protocols.

### 4.1 Future perspectives

Advanced MR images acquired during surgery could gain insight into the effect of mechanical manipulation of a child’s brain. Data from these sequences could be useful for research into biomarkers predicting surgery-induced early effects of intraoperative complications (Choudhri et al., 2015; Metwali et al., 2020). For example, in research on surgery-induced cerebellar mutism syndrome (CMS), intraoperative dMRI and ASL imaging might help find biomarkers that could be used for treatment development and prevention strategies (Ahmadian et al., 2021). In such a way, CMS-related diffusion abnormalities that have been seen in the proximal efferent cerebellar pathways (Avula et al., 2014; Keil et al., 2018; Avula, 2020), could be detected earlier on by means of multi-shell fiber tractography that is more anatomically accurate. Also, the onset of supratentorial cortical hypoperfusion related to CMS could be detected earlier on intraoperative ASL perfusion maps (Ahmadian et al., 2021). Hypoperfusion in this region was previously found to result from cortico-cerebellar diaschisis (Keil et al., 2018).

Another example is the prediction of early postoperative seizures after supratentorial brain tumor surgery with intraoperative ASL and APTw imaging. These seizures have been associated with hemorrhage in the resection cavity (Chassoux and Landre, 2017; Samudra et al., 2019; Ersoy et al., 2020). As the onset of seizures in children has been associated with cortical hyperperfusion (Oishi et al., 2012; Keil et al., 2018), intraoperative perfusion imaging with ASL might be an early predictor for seizures and even lead to intraoperative monitoring and prevention (Palaniswamy et al., 2019). APTw images could have a similar effect due to the association of seizures with a reduced pH that could be picked up by a reduced APTw signal (Magnotta et al., 2012; Jin et al., 2017). However, this seizure-induced change in pH change was said to be smaller than the precision of the pH measurement derived from APTw data (Jin et al., 2017). Nevertheless, intraoperative APTw images could have the unique potential to gain more insight into the physiologic processes of postoperative seizures in young children.

Reports focusing on the added value of intraoperative use of advanced MRI, particularly metabolic MRI, in the pediatric brain tumor population are scarce to date. The existing literature was often of a descriptive nature, and randomized controlled trials are lacking. Whilst we could learn from proof of concept reports that use advanced ioMRI in the adult population (Lindner et al., 2017), a specific investigation into the pediatric population is required to understand this unique situation better. Further, the majority of the included studies of our systematic literature search originated from the same clinical center (Abernethy et al., 2012; Yousaf et al., 2012; Avula et al., 2013; 2021; Tejada et al., 2018; Sunderland et al., 2021). Their findings could therefore potentially represent overlapping patient data.

To study the effect of surgery on a child’s brain, a multiparametric approach, including diffusion, perfusion, and metabolic ioMRI could be useful. Especially with the opportunity of tissue pathological validation on the spot. Recent developments regarding *in vivo* microscopy and high-speed histopathological diagnostics (Hollon et al., 2020) may facilitate immediate validation of these advanced ioMRI sequences in the near future.

Further development of accelerated imaging techniques could also be explored to reduce the acquisition time or to improve image quality. Particularly image acceleration techniques that could be used with the limited number of single-loop receiving coils that are currently available in the intraoperative setting. Alternatively, recent hardware development of thinner, more flexible, or multi-channel coils could improve image quality and reduce the transition time to and from the ioMRI suite.

The application of advanced ioMRI could be potentially valuable in providing new relevant information of the brain in the peroperative setting. The implementation might be challenging and involves close collaboration between neurosurgeons, neuroradiologists, and physicists. Collaboration among professionals from different ioMRI centres will contribute and support progress in this field.

## 5 Conclusion

The potential added value of advanced MRI in the intraoperative setting for pediatric brain tumors is to prevent damage to important structures, to provide additional physiological or metabolic information, or to indicate the onset of postoperative changes. Current developments within various advanced ioMRI sequences are promising with regard to providing in-depth tissue information.
